# Nutrition, Choice and Health-Related Claims

**DOI:** 10.3390/nu12030650

**Published:** 2020-02-28

**Authors:** Tiziana de-Magistris

**Affiliations:** 1 Unidad de Economía Agroalimentaria y de los Recursos Naturales—Centro de Investigación y Tecnología Agroalimentaria de Aragón (CITA), Gobierno de Aragón, 50004 Zaragoza, Spain; tmagistris@gmail.com; 2 Instituto Agroalimentario de Aragón, IA2 (CITA-Universidad de Zaragoza), 50009 Zaragoza, Spain

Scientific evidence shows that food consumption is one of the main causes that increases the risk of developing a non-communicable disease (NCD). One of the mechanisms introduced to ensure more informed food purchases that lead to healthier diets is the introduction in the marketplace of functional food products to provide information on the nutritional and health properties that certain foods possess. This information is transmitted to consumers via different nutritional and health claims. 

Two studies investigated the prevalence of front-of-package (FoP) claims in Brazilian packaged food. Duran et al. [1] found that nutritional claims (NCs) were the most prevalent, followed by health claims (HCs), especially in breakfast cereals and dairy beverages. Zancheta Ricardo et al. [2] examined the presence of trans fat information on the nutrition facts panel. The authors reported that 81.3% of the 11,434 products analyzed, did not present a source of trans fats in the list of ingredients. However, bakery products, cookies and crackers, candies and desserts, snacks, and convenience foods had the highest percentages of trans fat claims. 

Two studies explored the context of foods and drinks with healthy and nutritious attributes in the United Kingdom (UK). Cesar Revoredo-Giha et al. [3] indicated that trading down in quality occurs in most of the studied categories and countries, and when households trade down, they moved to products with worse nutritional qualities. Likewise, Monserrat and Revoredo-Giha [4] assessed to what extent health and nutrition claims made by breakfast cereals had an impact on their market success. 

Four studies focused on consumers’ preferences and willingness to pay (WTP) for functional food products, while one study examined the role of functional food in disease prevention. Vischeccia et al. [5] analyzed a mozzarella cheese carrying reduced fat and enriched in omega-3 claims. The authors found that consumers´ willingness to pay for health claims was higher than nutrition claims and that naturally enriched omega-3 was the most preferred claim. Castellari et al [6] indicated that providing new nutritional information significantly increased the WTP for a jam-like fruit compote enriched with aloe vera gel. Panea and Ripoll [7] investigated the perception of pork quality that consumers attached to the functional combination of the addition of extracts derived from plants (pork-derived extracts added to pork feed) and the meat conservation conditions (packaging and time exposure). Likewise, Verneau et al. [8] analyzed the effect of information about the health benefit produced by lycopene on the WTP for canned crushed tomatoes enriched with lycopene. The results showed a relevant impact of information on WTP in the case of lycopene-enriched products. Finally, Plasek et al. [9] examined the role of functional foods in disease prevention in Hungary on about 13 diseases with four prevention methods. The results reported that functional foods prevented digestive problems, a weakened immune system and a high cholesterol level. 

Two studies investigated consumers´ preferences for food products carrying sustainable and nutritional labels simultaneously. Almli et al. [10] observed a broad heterogeneity in health attitudes among Norwegian, Romanian, and Turkish organic consumers. Akaichi et al. [11] showed that consumers did not view desirable food attributes (such as organic, local, fair trade and high animal welfare) as unrelated to health-related food labels.

Two lines of investigation analyzed how consumers ranked different nutritional claims. Gracia and Barreiro-Hurlé [12] reported that the ranking of claims differs between biscuits and pastries and across consumers. Moreover, the results indicated that, for the average consumer, the most important nutritional claims for the two cereal products were “reduced saturated fat” and “with no added sugar”. On the other hand, the least important claim was “low salt”. Annunziata and Mariani [13] examined six specific claims on the basis of a web survey carried out on a sample of 504 consumers. Findings revealed that there is little attention paid to nutritional health claims and their use is not widespread. 

Two studies explored consumers’ choices for health information. Sogari et al. [14] tested the impact of labeling wholegrain pasta with a health message descriptor displayed at the point-of-purchase (POP) on consumer choice in a campus dining setting. Findings indicated that only the message about vitamin benefits had a significant effect on this choice, with a higher probability of selecting this pasta than the no-message condition and also a higher probability than the fiber message condition. Sajdakowska and Tekień [15] determined different segments of consumers based on their preferences towards some statements related to nutrition presented on a yoghurt label with a precise focus on aspects of the increased and decreased content of some ingredient.

Four studies focused on psychological factors affecting consumers’ preferences for NCs. Guzek et al. [16] determined the influence of food neophobia (FN) about allergens on the food product choices. The respondents characterized by a high level of FN less commonly chose dishes characterized by neophobic potential as a starter (carpaccio), main course (risotto ai frutti di mare) and dessert (zabaglione). Benson et al. [17] identified knowledge as the key factor influencing how much individuals believe nutritional claims and their perceptions. López-Galán and de-Magistris [18], assessed whether an emotional eating style influenced the purchase of food products carrying these claims. Findings of this study suggested that emotional eating negatively impacts purchasing behavior related to nutritional claims. Finally, Bazzani et al. [19] showed that health consciousness was an important driver in the use of wine labels, such as clean labels and alcohol content.

The last two studies investigated new determinants influencing purchase intention for functional foods. Berhaupt-Glickstein et al. [20], investigated the effects of health claims carried by green tea on purchase intentions among adults 55 years of age and older living in the US. Factors that mitigated the claim’s effects on purchase intentions were: race/ethnicity, age, importance of health claims, supplement use, health, worry about health/becoming sick with cancer, worry that led to dietary change, green tea consumption and the relationship between green tea and cancer. Finally, Temesi et al. [21] revealed that perceived fit of the carrier and the ingredient is a major determinant of purchase intention, together with health concerns and attitudes to functional foods.

The present Special Issue focused on the role of nutritional properties and/or health-related claims of food products and functional food products on choice preferences, choice behavior, healthy eating/healthy diet and the willingness to pay for certain foods. 
